# IS ETHNIC HERITAGE MEANINGFUL IN ADULTHOOD? ETHNIC IDENTITY AND WELL-BEING IN MEXICAN-ORIGIN FEMALE ADULTS

**DOI:** 10.1093/geroni/igac059.1914

**Published:** 2022-12-20

**Authors:** Hin Wing (Florence) Tse, Jiaxiu Song, Elizabeth Muñoz, Su Yeong Kim

**Affiliations:** Department of Human Development and Family Sciences, The University of Texas at Austin, Austin, Texas, United States; The University of Texas at Austin, Austin, Texas, United States; The University of Texas at Austin, Austin, Texas, United States; The University of Texas at Austin, Austin, Texas, United States

## Abstract

Being the largest immigrant group in the U.S., the process of exploring, forming, and retaining ethnic identity is a critical component to Mexican immigrants’ successful adaptation and wellbeing. Despite recognizing the dynamic nature of ethnic identity development, extant research has predominantly focused on adolescence, overlooking the development and impact of ethnic identity in adulthood, when individuals’ experiences are continuously shaped by critical life events (e.g., moving to a new country). Additionally, there is a lack of longitudinal research on mid-late adulthood. Filling these gaps, this study utilized a three-wave longitudinal dataset of 595 Mexican-origin female adults (Mage.wave1 = 38.39) to examine their initial levels and trajectories of ethnic identity development (i.e., exploration, centrality, and resolution) and understand how these individuals’ initial levels and trajectories of ethnic identity are associated with their wellbeing (i.e., life meaning, resilience, and depressive symptoms) at Wave 3. Using latent growth curve modeling, unconditional models revealed that initial levels of ethnic identity in Mexican-origin female adults were moderately high, and that their centrality and resolution of ethnic identity remained stable while their exploration of heritage identity increased over time. The conditional model also showed that Mexican-origin female adults’ higher initial levels of centrality and resolution were associated with a greater sense of life meaning (and resiliency, only true for resolution levels) but not with depressive symptoms. These findings suggest that ethnic heritage is associated with more positive perception in life and may inform interventions on developing a positive ethnic identity that is related to better wellbeing.

